# Factors associated with PTSD symptoms and quality of life among nurses during the COVID-19 pandemic: A cross-sectional study

**DOI:** 10.1371/journal.pone.0283500

**Published:** 2023-03-24

**Authors:** Cian-Cian Lin, Chin-Bin Yeh

**Affiliations:** Department of Psychiatry, Tri-Service General Hospital, National Defense Medical Center, Taipei, Taiwan; Al-Jouf University College of Pharmacy, SAUDI ARABIA

## Abstract

**Background:**

Healthcare workers, especially nurses, were one of the most vulnerable groups for developing posttraumatic stress disorder (PTSD) during the coronavirus disease 2019 (COVID-19) pandemic, which also affected their quality of life. However, only limited research has investigated the individual psychological factors as well as the environmental factors responsible for these effects of the pandemic. Demoralization is a state of loss of meaning and anhedonia, which we thought to be an important mediator between fear and PTSD among frontline nurses during the pandemic. This study aimed to explore the role of demoralization in the mechanisms of posttraumatic stress symptoms of nurses facing different infection risks and influencing factors on their well-being.

**Method:**

A cross sectional study was conducted from September 16, 2021 to October 8, 2021 in a medical center in Northern Taiwan. Online questionnaires were used to collect data, including age, sex, vaccination status, working years, previous quarantine experiences, psychiatric history, traumatic events and scales for measuring fear of COVID-19, demoralization, symptoms of posttraumatic stress, depression, anxiety and stress, burnout level, teamwork performance and quality of life. Hierarchical regression analysis and mediation analysis were utilized to identify associated risk factors and mechanisms.

**Result:**

Among 351 included nurses, 148 worked in high-risk areas directly exposed to COVID-19 patients or patients with respiratory symptoms, while 203 nurses worked in low-risk areas. Overall, nurses in the low-risk group had greater fear of COVID-19, and greater demoralization and burnout level, along with poorer teamwork and quality of life. Demoralization was found to have mediating effect in both the high-risk group and low-risk group on the relationships between fear of COVID-19 and posttraumatic stress symptoms. Levels of burnout and teamwork may serve as mediators between depression, anxiety, stress and quality of life.

**Conclusion:**

Hospital-based nurses appear to be at high risk for developing posttraumatic stress disorder during the COVID-19 pandemic. Study findings demonstrated specific associated factors that should be the focus of nursing administration and hospital management while employing preventive measures, psychological resilience of nurses or systematic managements. Future longitudinal research is needed to improve management in pandemic conditions.

## Introduction

### The COVID-19 pandemic in Taiwan

The coronavirus-19 pandemic was declared as a global pandemic on 30^th^ January 2020 by World Health Organization (WHO). Since then, the COVID-19 outbreak has posed tremendous threats to various industries and healthcare aspects worldwide. A total of 497 million deaths had been reported worldwide by October 2021 [1]. In contrast, the virus was almost held at bay in Taiwan during the first year of the outbreak due to applying preventive measures in a timely manner [2]. The large-scale community surge did not erupt until May 2021 in Taiwan [3]. The Central Epidemic Command Center (CECC) raised the epidemic level once to 3 (level 4 resulted in lockdown) between May 15, 2021 and July 26, 2021.

### PTSD risk among nurses in the COVID-19 pandemic

The pandemic has caused negative psychological impacts around the world. Posttraumatic stress disorder (PTSD) has conventionally been a focus of concern following major traumatic events. PTSD prevalence during public health disasters of the general population was reported to be about 19.9% after the Severe Acute Respiratory Syndrome (SARS) outbreak in 2003, and 13.7% after H1N1 influenza A pandemic (2009) or Middle East Respiratory Syndrome (MERS) in 2012 [4]. Some groups were more susceptible than others to psychological stress during the pandemic, including healthcare professionals, people with previous traumatic event exposure, people with pre-existing psychiatric history, COVID-19 survivors or those who were quarantined [4, 5]. Increased risk for PTSD was noted in SARS survivors (25.6%), especially those who were healthcare workers (40.7%) in a Hong Kong survey [6]. COVID-19 was no exception PTSD prevalence was 26.9% in healthcare workers within 12 months post-pandemic, which was higher than the rate of the general population (19.9%) [4]. Several studies showed that healthcare workers providing direct care to confirmed cases had a particularly higher trend of overall PTSD prevalence than non-frontline workers [4]. Nurses especially bore a higher risk compared with physicians, suggesting the nurses perceived more psychological burdens [7, 8].

Previous investigations examined the predisposing, precipitating or resilience factors of PTSD. For example, the predisposing factors of PTSD include genetic loading, early traumatic experiences, and prior maladaptive cognitive schema [9, 10]. The precipitating factors include different trauma types and levels of severity. Good resilience factors include active coping style, seeking social support or adequate cognitive flexibility [11]. Risk factors for PTSD in nurses have been reported to include demographic factors such as younger age and female gender [12, 13]. Whether or not vaccination would be able to reduce mental distress remained debatable [14, 15]. However, fear of contagion or death, worry about lack of personal protective equipment (e.g., masks, face shields) in the pandemic have been described universally as risk factors for PTSD [16, 17].

### The relationship between demoralization and PTSD

A psychological state called demoralization, may possibly develop when facing existential distress with uncertain duration as in epidemic or pandemic situations. It features a sense of hopelessness, helplessness and loss of the meaning of life. The syndrome was first reported by Kissane et al. (2001) [18]. Demoralization was initially used to describe losing the meaning of life and having a sense of hopelessness after facing persistent stress without any possible resolution, particularly a major illness or cancer. Prior study has shown that demoralization may predict PTSD [19]. A bidirectional relationship between demoralization and PTSD has even been suggested. Demoralized patients tend to demonstrate avoidance, which is a PTSD symptom manifested as a coping strategy while PTSD patients may develop loss of meaning when they face difficulties dealing with PTSD symptoms [20, 21]. Fear itself may cause a sense of helplessness, which may predict subsequent PTSD [22]. One study also indicated increased feelings of disheartenment during the pandemic among a group of medical students [23]. However, posttraumatic growth is expected for better mental outcomes in the aftermath of major trauma. Demoralization was found to negatively influence posttraumatic growth in a previous study from Taiwan that focused on 200 cancer patients [24]. Retaining the meaning of life was said to be positively associated with posttraumatic growth in the general population [25, 26]. PTSD consists of interactions between one’s emotions and cognition. Therefore, whether PTSD develops after a trauma exposure is mostly dependent upon the deficits in memory processing and cognitive interpretation [27]. Either the posttraumatic growth or the process of developing PTSD may be influenced by demoralization. Therefore, we hypothesized that demoralization may have an essential role in the relationship between fear of COVID-19 and PTSD.

### The relationship between and Teamwork performance and quality of life

Besides having a higher prevalence of PTSD, nurses’ quality of life has also been severely affected amid the pandemic. Systematic confounding factors including increasing workload, higher perceived burnout level, and teamwork challenges when facing unprecedented scenarios have all been suggested to be associated with reduced quality of life [28, 29]. Teamwork capacity would thus be a potential protective factor for nurses’ quality of life. From an administrative perspective, nurse’s poor quality of life can present a huge challenge to hospital managers and may ultimately compromise patients’ safety. Human resources in the hospital were particularly indispensable during the outbreak. Discovering factors that influenced employment maintenance during COVID-19 was especially helpful to hospital and nursing administration.

### Aims of the study

Though previous studies have surveyed the epidemiology of PTSD and nurses’ well-being during the COVID-19 pandemic, they seldom discussed about mechanisms behind the development of PTSD. Therefore, the primary purposes of this study were to investigate the relationship between fear and PTSD and the role of demoralization as the mediator between these factors (S1 Appendix in S1 File). We hypothesized that “fear” could be associated with PTSD symptoms and that demoralization may mediate this relationship. The second study aim was to explore the relationship between anxiety/depression and impaired quality of life (S1 Appendix in S1 File), for which teamwork performance or burnout level may play a role between them.

## Methods

### Study design and sample

**Fig 1:**
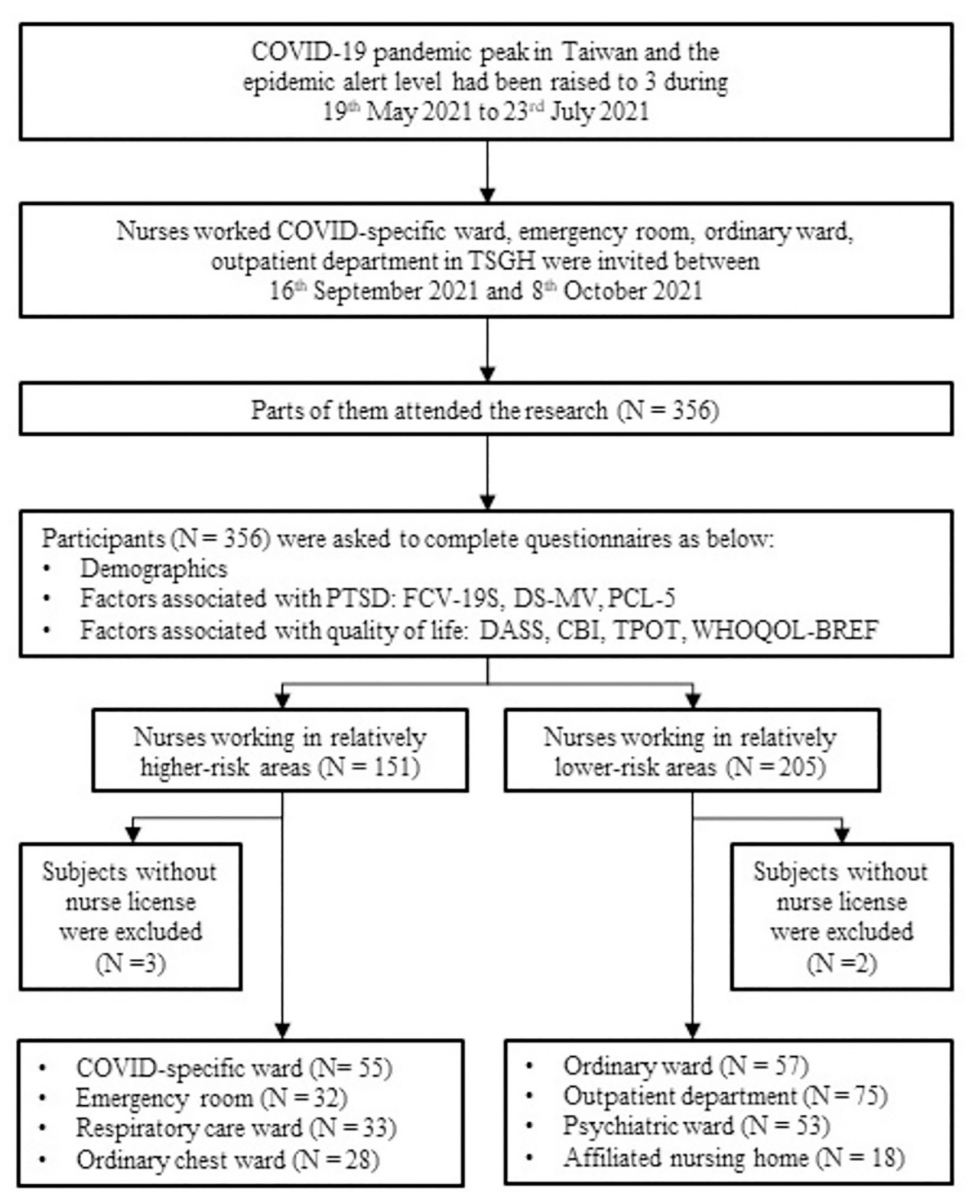
Recruitment and participation of the study.

This cross-sectional survey actively recruited licensed nurses working in a university affiliated hospital in Taipei, Taiwan, between September 16 2021 and October 8 2021. Among 1600 nurses working in the hospital, about 345 had worked in high-risk areas defined as COVID-specific ward, emergency room, respiratory care ward or ordinary chest ward at the peak of the pandemic. Using convenience sampling, nurses were recruited from high-risk areas (N = 151; COVID-specific ward = 55, emergency room = 32, respiratory care ward = 33, and ordinary chest ward = 28); and from low-risk areas (N = 205; ordinary ward = 57, outpatient department = 75, psychiatric ward = 53, and hospital-affiliated nursing home = 18). Three high-risk groups and 2 low-risk groups were also found to include other types of healthcare providers without nursing licenses and therefore were excluded. Ultimately 351 nurses were enrolled (Fig 1).

### Instruments

Questions about lifestyle change, vaccination status, quarantine experience, traumatic experience during the pandemic and seven self-reported validated questionnaires were applied in the questionnaire for all enrolled subjects. Measures of factors hypothesized to be associated with PTSD included the following instruments: Fear of COVID-19 Scale (FCV-19S), Demoralization Scale—Mandarin Version (DS-MV), Posttraumatic Stress Disorder Checklist for DSM-5 (PCL-5). Surveys associated with quality of life included Depression Anxiety Stress Scale (DASS), World Health Organization Quality of Life-BREF (WHOQOL-BREF), Copenhagen Burnout Inventory (CBI), and Team Performance Observation Tool (TPOT) (Fig 1).

#### Fear of COVID-19 Scale (FCV-19S)

This seven-item scale, scored on a five-point Likert scale, was developed in 2020 for evaluation of fear of COVID-19. Questions addressed mood changes, and cognition or somatic complaints after exposed to the pandemic [30]. Cronbach’s α was 0.92, which was consistent with previous study results (Cronbach’s α = 0.87), indicating satisfactory internal consistency [30].

#### Demoralization Scale (DS-MV)

The Demoralization Scale with 24 items has been used for decades to measure the five core dimensions proposed by Kissane et al., including loss of meaning, dysphoria, disheartenment, helplessness and sense of failure [18]. It has been translated into several languages and widely utilized all over the world. The Mandarin version, translated by Fang et al., is rated on a five-point Likert scale ranging from 1 (strongly disagree) to 5 (strongly agree). Good internal consistency reliability (Cronbach’s α = 0.928) was also found in a previous study (Cronbach’s α = 0.928) [31]. The cut-off point was 30.

#### Posttraumatic Stress Disorder Checklist for DSM-5 (PCL-5)

PCL-5 is a highly utilized self-administered measurement of PTSD that helps with screening or making provisional diagnoses [32, 33]. Cronbach’s α was 0.979, indicating good reliability as also noted in the previous Chinese version (0.957) [32]. The scale contains 20 items corresponding to the PTSD symptoms in the Fifth edition of the Diagnostic and Statistical Manual of Mental Disorders (DSM-5). Each of the items is scored by a five-point Likert scale that represents the distress level of symptoms’ severity, ranging from 0 (not at all) to 4 (extremely). The cut-off point was 31.

#### Depression Anxiety Stress Scale (DASS)

The 21-item DASS questionnaire screens for depression, anxiety and stress level. The depression subscale measures hopelessness, low self-esteem and low positive affect. The anxiety subscale evaluates somatic symptoms, including automatic arousal. The stress subscale is a four-point Likert scale, with 0 representing “never” and 3 representing “almost always”; it evaluates individuals’ tension and negative affect [34]. Good internal consistency was demonstrated (Cronbach’s α = 0.962) as also reported previously (Cronbach’s α ranging from 0.82 to 0.97) [35].

#### World Health Organization Quality of Life-BREF (WHOQOL-BREF)

This 28-item instrument measures four domains of quality of life, including physical health, psychological health, social relationships and environmental health. Each of the items is scored on a 1 (not at all) to 5 (extreme amount) Likert scale and is transformed to a 0–100 scale. The internal consistency (Cronbach’s alpha) coefficient was 0.941, which was higher than that in the psychometrics of a previous study for the four domains ranging from 0.70 to 0.77 [36].

#### Copenhagen Burnout Inventory (CBI)

The inventory consists of 21 items. Four dimensions of burnout were investigated in the Taiwanese version translated by Yeh et al, 2007, which included: personal-related burnout, work-related burnout, client-related burnout and overcommitment. The Cronbach’s α was 0.965, higher than that in a previous study (four subscales were all more than 0.84), indicating good internal consistency. Each item is rated from 0 (never)– 4 (always) five-point Likert scale [37].

#### Team Performance Observation Tool (TPOT)

This five-point Likert scale (1 = strongly disagree, 5 = strongly agree) was derived from the Agency for Healthcare and Research Quality (AHRQ), USA. It was developed to assess the teamwork capacity of a clinical team categorized into five domains, including team structure, communication, leadership, situation monitoring and mutual support. The related capacity was also introduced by the Joint Commission of Taiwan. Cronbach’s α was 0.975, indicating good reliability, which was consistent with that of a previous study (Cronbach’s α = 0.921) [38].

#### Procedure

Questionnaires were administered between September 16, 2021 and October 8, 2021. Several restrictions were adopted during that wave of the pandemic, including limits on the number of people allowed in a given space, not being allowing to travel abroad, shortage of manpower at the hospital and some nurses having to work longer times at the hospital, and shortage of protective equipment for nursing staff. The link to the questionnaires via Google Forms was provided as a quick response code to reduce contact and the risk of contagion. The study purpose was explained to the subjects, along with their associated rights and nurses were given informed consent forms in paper form. Online questionnaires were filled out by smartphones. The study protocol was reviewed and approved by the Institutional Review Board of Tri-Service General Hospital, Taipei, Taiwan. All participating nurses provided signed informed consent to participate in the study.

### Statistical analysis

All statistical analyses were performed using SPSS v.22. (IBM, Armonk, NY, USA). Chi-squared test and independent t test were performed to examine differences between nurses’ groups in age, sex, work experience, other socio-demographics characteristics and the mean scores of the seven scales and subscales. Binary logistic regressions on characteristics in categorical data separately were utilized with dependent variable as PCL-5≧31 to screen for the possible risk factors of PTSD. Hierarchical regression was conducted in three levels to identify the individual four effects of age, having previous major traumatic experience, fear of COVID-19 and DS-MV (Demoralization Scale—Mandarin version) on PTSD (PCL-5≧31) and the impacts of fear of COVID-19 and DS-MV after controlling for baseline demographics, since these were factors relatively specific to individual experiences.

Mediation analysis was carried out with the independent variable (fear of COVID-19 scale) as the total score of the fear of COVID-19 scale and the dependent variable (Posttraumatic Stress Disorder Checklist for DSM-5) as the total score of Posttraumatic Stress Disorder Checklist for DSM-5 (PCL-5). Single mediator (Demoralization Scale) with total score of the Demoralization Scale was examined in the high-risk group and low-risk group of nurses. A two-mediator (Depression, Anxiety and Stress Scale and WHOQOL-BREF) model seeking to explore the impacts of total scores of the Depression, Anxiety and Stress Scale (DASS) on WHOQOL-BREF transmitted through total scores of Copenhagen Burnout Inventory (CBI) and Team Performance Observation Tool (TPOT) were estimated as well. Sobel’s test was calculated for verification. Mediation ratio (P_M_), defined as the ratio of the indirect effect to the total effect, was also assessed in each mediation model.

## Results

### Demographics and statistics

Sociodemographic characteristics including age, sex, work experience, lifestyle change, whether identified as potential contact, family member confirmed COVID-19, family member died from COVID-19, work from home or workplace closed, vaccinated doses, received vaccine types, quarantine experience, traumatic experiences during COVID-19, previous psychiatric history, previous major traumatic events are presented in Table 1. The majority of the subjects were female (94.87%), with mean age 33.83, 10.25 years of nursing experience and already had two COVID-19 vaccinations (95.44%). Chi-squared test identified whether nurses of the high-risk group had much more experience working from home or workplace closed (N = 48, *x*^2^ = 5.12, *p* = 0.024), received Taiwan domestic vaccine (N = 4, *x*^2^ = 5.55, *p* = 0.018), quarantined in COVID-specific ward (N = 8, *x*^2^ = 8.27, *p* = 0.004) and had witnessed patients’ deaths (N = 18, *x*^2^ = 19.9, *p*<0.001) or patients’ lives being threatened (N = 35, *x*^2^ = 26.85, *p*<0.001).

**Table 1:** Comparison of demographics of nurses in high-risk and low-risk groups.

	Total (N = 351)	High risk group (N = 148)	Low risk group (N = 203)	Statistics
	*N (%) or M* ± *SD*	*N (%) or M* ± *SD*	*N (%) or M* ± *SD*	*p*
**Sociodemographic characteristics**				
**Age ≧ 30-year-old (N)**	188 (53.56%)	74 (50.00%)	114 (56.16%)	*p* = 0.253
**Gender (N)**				*p* = 0.490
Female	333 (94.87%)	139 (93.92%)	194 (95.6%)	
Male	18 (5.13%)	9 (6.08%)	9 (4.4%)
**Working experience ≧ 10 years (N)**	161 (45.87%)	63 (42.57%)	98 (48.28%)	*p* = 0.289
**Lifestyle change**				
Social distancing (N)	328 (93.45%)	139 (93.24%)	189 (93.10%)	*p* = 0.760
Face mask wearing (N)	345 (98.29%)	147 (99.32%)	198 (97.54%)	*p* = 0.202
Frequent handwashing (N)	327 (93.12%)	139 (93.92%)	188 (92.61%)	*p* = 0.632
**Identified as potential contact (N)**	73 (20.80%)	34 (22.97%)	39 (19.21%)	*p* = 0.391
**Work from home or workplace closed (N)**	92 (26.21%)	48 (32.43%	44 (21.67%)	*p* = 0.024*
**Family member confirmed COVID-19 (N)**	6 (1.71%)	2 (1.35%)	4 (1.97%)	*p* = 0.659
**Family member died from COVID-19 (N)**	3 (0.85%)	0 (0%)	3 (1.48%)	*p* = 0.137
**COVID-19 vaccine shots**				*p* = 0.436
Had two doses of vaccines (N)	335 (95.44%)	142 (95.95%)	193 (95.07%)	
Had one dose of vaccine (N)	11 (3.13%)	3 (2.03%)	8 (3.94%)	
Not vaccinated yet (N)	5 (1.42%)	3 (2.03%)	2 (0.99%)	
**Received COVID-19 vaccine types**				
AstraZeneca (N)	246 (70.09%)	106 (71.62%)	140 (68.97%)	*p* = 0.622
Moderna (N)	176 (50.14%)	70 (47.30%)	107 (52.71%)	*p* = 0.439
Taiwan domestic vaccine (N)	4 (1.13%)	4 (2.70%)	0 (0%)	*p* = 0.018*
BNT (N)	1 (0.28%)	1 (0.68%)	0 (0%)	*p* = 0.241
Mixed shots with AZ & Moderna (N)	78 (22.22%)	32 (21.62%)	46 (22.66%)	*p* = 0.817
Mixed shots with Moderna & Taiwan domestic vaccine (N)	4 (1.14%)	4 (2.70%)	0 (0%)	*p* = 0.018*
**Quarantine experience**				
Quarantined (N)	39 (11.11%)	22 (14.86%)	17 (8.37%)	*p* = 0.056
Quarantined in COVID specific ward (N)	9 (2.56%)	8 (5.40%)	1 (0.94%)	*p* = 0.004**
Quarantined in ordinary ward (N)	9 (2.56%)	6 (4.05%)	3 (1.48%)	*p* = 0.132
Home quarantined (N)	21 (5.98%)	8 (5.40%)	13 (6.40%)	*p* = 0.697
**Traumatic experiences during COVID-19**				
I witnessed patients’ death with COVID-19 (N)	20 (5.70%)	18 (12.16%)	2 (0.99%)	*p* <0.001***
I witnessed patients’ lives threatened (N)	45 (12.82%)	35 (23.65%)	10 (4.93%)	*p* <0.001***
Faced life-threatening conditions on myself (N)	2 (0.57%)	1 (0.68%)	1 (0.94%)	*p* = 0.822
My family faced life-threatening conditions (N)	3 (0.85%)	1 (0.68%)	2 (0.99%)	*p* = 0.756
**Had previous psychiatric history (N)**	23 (6.55%)	7 (4.73%)	16 (7.88%)	*p* = 0.239
**Had previous major traumatic event (N)**	26 (7.41%)	11 (7.43%)	15 (7.39%)	*p* = 0.988
**Psychometric instruments**				
**DASS**				
Depression subscale	6.78 ± 8.28	6.20 ± 7.93	7.19 ± 8.53	*p* = 0.270
Anxiety subscale	6.59 ± 7.68	6.37 ± 7.85	6.75 ± 7.56	*p* = 0.644
Stress subscale	9.89 ± 9.25	9.03 ± 9.02	10.52 ± 9.39	*p* = 0.135
Total score	23.25 ± 23.96	21.60 ± 23.74	24.46 ± 24.11	*p* = 0.269
**PCL-5**	12.95 ± 15.65	11.30 ± 15.51	14.16 ± 15.69	*p* = 0.091
**FCV-19S**	2.40 ± 0.86	2.28 ± 0.89	2.48 ± 0.83	*p* = 0.035*
**WHOQOL-BREF**				
Dom 1 (Physical health)	13.82 ± 2.22	14.14 ± 2.27	13.59 ± 2.16	*p* = 0.021*
Dom 2 (Psychological health)	12.40 ± 2.56	12.82 ± 2.63	12.09 ± 2.47	*p* = 0.008**
Dom 3TW (Social relationships)	13.15 ± 2.40	13.22 ± 2.44	13.10 ± 2.37	*p* = 0.632
Dom 4TW (Environmental health)	13.04 ± 2.35	13.38 ± 2.39	12.79 ± 2.29	*p* = 0.019*
Total score	52.41 ± 8.38	53.57 ± 8.65	51.57 ± 8.09	*p* = 0.027*
**CBI**				
Personal burnout	41.14 ± 23.24	37.74 ± 21.96	43.62 ± 23.88	*p* = 0.019*
Work-related burnout	37.32 ± 24.55	34.53 ± 24.53	39.36 ± 24.42	*p* = 0.068
Client-related burnout	35.75 ± 24.59	31.36 ± 24.12	38.96 ± 24.48	*p* = 0.004**
Overcommitment	32.79 ± 23.12	30.74 ± 22.96	34.29 ± 23.18	*p* = 0.157
Total score	36.70 ± 20.95	33.49 ± 20.56	39.05 ± 20.97	*p* = 0.014*
**DS-MV**	30.19 ± 14.05	26.99 ± 13.81	32.53 ± 13.79	*p* <0.001***
**TPOT**				
Team structure	3.69 ± 0.74	3.83 ± 0.69	3.58 ± 0.75	*p* = 0.001**
Communication	3.74 ± 0.83	3.81 ± 0.85	3.69 ± 0.81	*p* = 0.191
Leadership	3.78 ± 0.70	3.87 ± 0.70	3.71 ± 0.70	*p* = 0.043*
Situation monitoring	3.92 ± 0.64	4.02 ± 0.62	3.84 ± 0.65	*p* = 0.010*
Mutual support	3.80 ± 0.75	3.87 ± 0.73	3.76 ± 0.77	*p* = 0.156
Total score	18.93 ± 3.08	19.40 ± 3.02	18.59 ± 3.10	*p* = 0.014*

AZ = AstraZeneca vaccine; DASS = Depression, Anxiety and Stress Scale; PCL-5 = PTSD Checklist for DSM-5; FCV-19S = The Fear of COVID-19 Scale; CBI = Copenhagen Burnout Inventory; DS-MV = Demoralization Scale-Mandarin Version; TPOT = Team Performance Observation Tool; *X*^2^ = Pearson’s chi-square

*p < 0.05,

**p <0.01,

***p <0.001

### Psychological and cognitive evaluations

The mean and standard deviation of scores of psychometric instruments including Depression, Anxiety and Stress Scale (DASS), PTSD Checklist for DSM-5 (PCL-5), The Fear of COVID-19 Scale (FCV-19S), World Health Organization Quality of Life-BREF (WHOQOL-BREF), Copenhagen Burnout Inventory (CBI), Demoralization Scale-Mandarin Version (DS-MV), Team Performance Observation Tool (TPOT) and their subscales are reported in Table 1. Mean scores of demoralization of all participating nurses were higher than the cutoff point derived from a prior survey of Australian cancer patients [39]. Mean score of fear of COVID-19 (2.40±0.86, *p* = 0.035), score of demoralization (30.19±14.05, *p*<0.001), total score of burnout (36.70±20.95, *p* = 0.014) and its scores of subscales of personal burnout (41.14±23.24, *p* = 0.019), client-related burnout (35.75±24.59, *p* = 0.004) were significantly more severe in the low-risk group. Also, nurses in the low-risk group reported poorer total score of WHOQOL-BREF (52.41±8.38, *p* = 0.027) and its scores of subscales of Domain 1 (physical health) (13.82±2.22, *p* = 0.021), Domain 2 (psychological health) (12.40±2.56, *p* = 0.008), Domain 4TW (environmental health) (13.04±2.35, *p* = 0.019), and lower total score of teamwork (18.93±3.08, *p* = 0.014) and its scores of subscales of team structure (3.69±0.74, *p* = 0.001), leadership (3.78±0.70, *p* = 0.043) and situation monitoring (3.92±0.64, *p* = 0.010).

### Regression analysis for PTSD predictors

Hierarchical regression was then performed at two levels for each of the possible predictors of PTSD in categorical measurements shown in Table 2, including age≧30 years, sex, working experience≧10 years, whether being quarantined or not, having traumatic experience during the pandemic, having previous psychiatric history, having previous major traumatic event, the fear of COVID-19 scale mean score>2.02 (mean score of New Zealand which was the lowest score across countries in 2020) and the demoralization scale (DS-MV) total score≧30 [30, 31]. As demonstrated in the second step, having previous major traumatic event (exp(β) = 2.12, *p*<0.001), FCV-19S mean score>2.02 (exp(β) = 1.34, *p* = 0.005), and DS-MV total score≧30 (exp(β) = 1.44, *p*<0.001) were positively associated with PTSD outcomes (Table 2).

**Table 2.** Predictors of PTSD (N = 351).

		PCL-5 score ≧ 31				R square
		B	Exp (B)	Wald	*P*	
**Step 1**	Age ≧ 30-year-old	0.81	2.24	1.93	*p* = 0.164	0.051
	Gender	-0.08	0.93	0.01	*p* = 0.928	
	Working experience ≧ 10 years	-0.17	0.84	0.09	*p* = 0.761	
	Quarantined	-0.17	0.84	0.10	*p* = 0.750	
	Had traumatic experiences during COVID-19	0.61	1.85	2.17	*p* = 0.140	
	Had previous psychiatric history	-0.40	0.67	0.31	*p* = 0.579	
	Had previous major traumatic event	1.68	5.36	12.91	*p* <0.001***	
**Step 2**	Age ≧ 30-year-old	0.94	2.57	2.32	*p* = 0.128	0.122
	Gender	-0.18	0.84	0.04	*p* = 0.838	
	Working experience ≧ 10 years	-0.39	0.68	0.43	*p* = 0.512	
	Quarantined	0.22	1.25	0.14	*p* = 0.707	
	Had traumatic experiences during COVID-19	0.64	1.91	2.14	*p* = 0.144	
	Had previous psychiatric history	-0.63	0.53	0.73	*p* = 0.392	
	Had previous major traumatic event	2.12	8.29	15.59	*p* <0.001***	
	FCV-19S > 2.02	1.34	3.82	7.80	*p* = 0.005**	
	DS-MV total score ≧ 30	1.44	4.24	11.07	*p* < 0.001**	

Hierarchical regression with PCL-5 score≧31 as dependent variable;

DASS = Depression, Anxiety and Stress Scale; PCL-5 = PTSD Checklist for DSM-5; FCV-19S = The Fear of COVID-19 Scale; CBI = Copenhagen Burnout Inventory; DS-MV = Demoralization Scale-Mandarin Version (cutoff point = 30) [31]; TPOT = Team Performance Observation Tool. The cut point of FCV-19S was set as 2.02 [30]

*p < 0.05,

**p <0.01,

***p <0.001

### Mediation of demoralization on the relationship between fear of COVID-19 and PTSD in high-risk (N = 148) and low-risk groups (N = 203)

**Fig 2:**
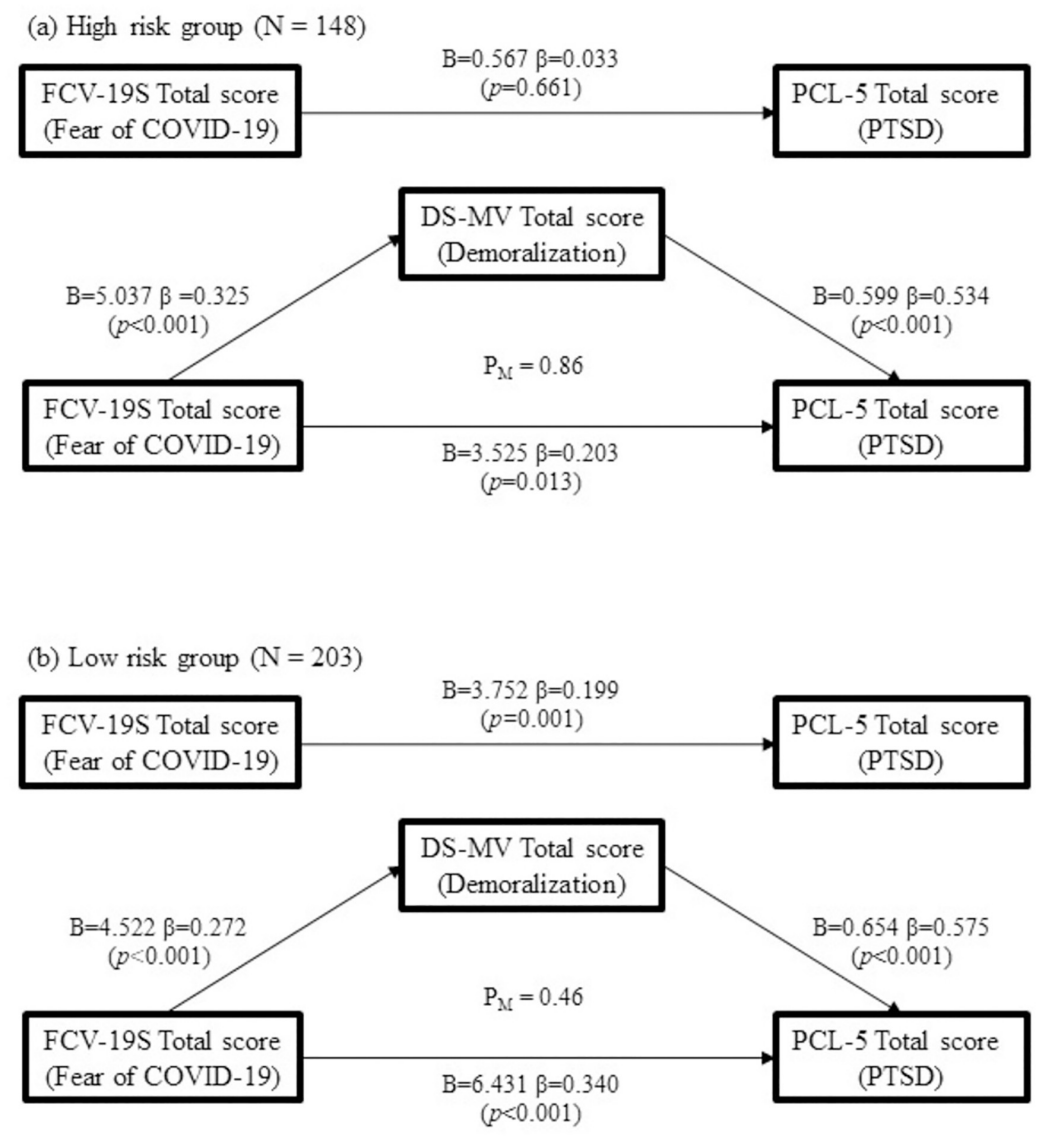
Mediation model of the effects of demoralization on the relationship between fear and PTSD in both (A) high-risk group (N = 148) (Sobel’s test: *Z* = 3.64, *p <* 0.001) and (B) low risk group (N = 203) (Sobel’s test: *Z* = 3.72, *p <* 0.001). (FCV-19S = The Fear of COVID-19 Scale; DS-MV = Demoralization Scale—Mandarin Version; PCL-5 = Posttraumatic Stress Disorder Checklist for DSM-5; P_M_ = the ratio of the indirect effect to the total effect).

Considering the significant differences between the two groups, demoralization was calculated as the mediator between fear of COVID-19 and PTSD by a mediation model in both high-risk group and low-risk group. Significant mediation was found in the high-risk group with *Z* value of Sobel’s test equaling 3.64, verifying the existence of mediating effect and a ratio of indirect effect to total mediation effect (P_M_) of 0.86 (Fig 2A) While the mediation effect in the low-risk group was from both the independent variable and the mediator, with *Z* value equaling 3.72 and P_M_ of 0.46 (Fig 2B).

### Parallel mediation effects of burnout and teamwork in the association between DASS and quality of life (N = 351)

**Fig 3:**
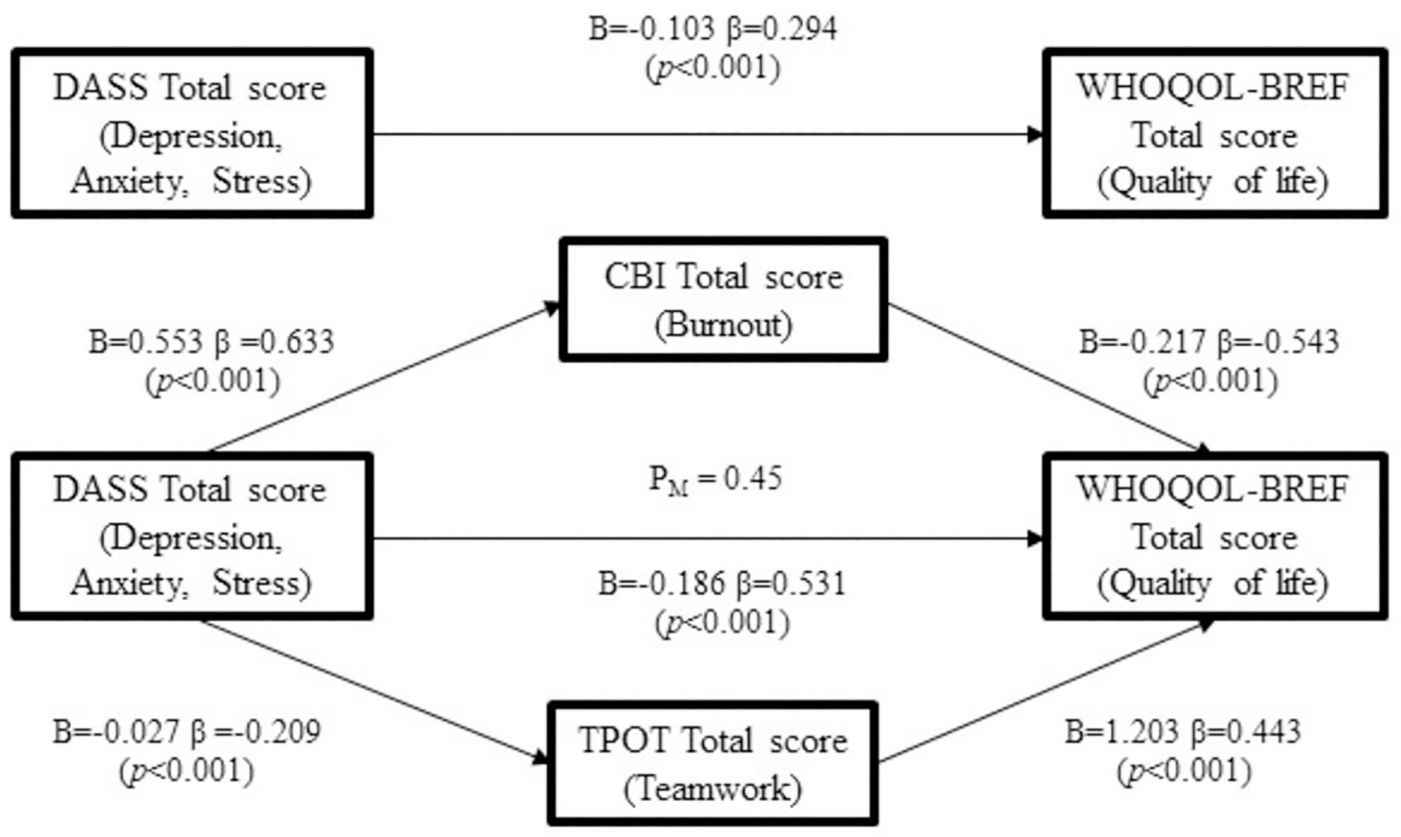
Mediation model of the effects of burnout and teamwork on the relationship between DASS and quality of life. (N = 351) (DASS = Depression, Anxiety, Stress Scale; CBI = Copenhagen Burnout Inventory; TPOT = Teamwork Performance Observation Tool; P_M_ = the ratio of the indirect effect to the total effect).

Scores of CBI and TPOT were examined as parallel mediators between DASS and WHOQOL-BREF since they were both statistically significant in the two separate paths. However, a significant correlation was found between the total scores of CBI and TPOT (β = -0.272, p < 0.001) even though the beta was not high (S2 Appendix in S1 File). The mediation model demonstrated the ability for burnout and teamwork to account for mediation effects of depression, anxiety, stress on quality of life with P_M_ of 0.45 (Fig 3).

## Discussion

To the best of our knowledge, this is the first study to evaluate PTSD symptoms and quality of life focusing only on nurses in Taiwan during the COVID-19 pandemic. Another survey showing high PTSD prevalence about 15% in Taiwan during the outbreak [40]. However, the study population included different types of healthcare workers with different duties and risks of exposure to the virus. Results showed that the high-risk group had higher rates of working from home or workplace closed, quarantined in COVID-specific ward, witnessed patient’s lives being threatened as expected compared with the low-risk group. The higher rate of receiving Taiwan domestic vaccine (i.e., Medigen vaccine), may be due to the small sample size. Since the first anti-COVID vaccination was August 23, 2021, which was months later, most healthcare workers received their vaccines (AstraZeneca and Moderna) months earlier in Taiwan. No differences were seen in either the high-risk or low-risk groups in sex, age and working experience. Lifestyle changes including maintaining social distance, wearing masks and frequent hand washing also had no differences since these measures were almost mandatory in Taiwan during the pandemic [41]. Nurses’ vaccination status presented no differences in rates of being identified as potential contact, family infected or family’s lives being threatened by the virus,. Compared with findings of other studies, the prevalence of previous psychiatric history was lower than prevalence of common mental disorders (6.55% vs. 17.1%) [42]. Prevalence of nurses with previous trauma event was also much lower than the previous lifetime rate reported in the Netherlands (7.41% vs. 71.7%) [43]. We speculated that the mental health conditions of healthcare workers may be better than that in general populations since health status was also a part of work requirements in hospitals.

In contrast to our expectations, the high-risk group of nurses did not show more psychological impacts than the low-risk group during the pandemic. The severity of PTSD symptoms demonstrated no significant differences between the two groups, even though the mean score of the whole study population was similar to the mean score (12.95 vs. 13.70–14.99) from a survey in Wuhan, China (N = 285). Contrary to results of the present study, which showed no significant differences in PTSD severity between the two groups, Johnson et al. reported that healthcare workers in Norway with direct contact with COVID-19 confirmed cases had more severe PTSD symptoms (N = 1773) [44]. On the other hand, the low-risk group showed a higher level of fear of COVID-19, and higher levels of demoralization, burnout, poorer quality of life and teamwork performance. These results may be associated with the time of sampling. An explanation of why high-risk group nurses had less fear of COVID-19 may be associated with their better preparation in terms of knowledge and training [45]. The mean total score of fear of COVID-19 of our population (total = 16.8; high-risk group = 15.96; low-risk group = 17.36 vs. 19.2) in a multicenter study of a Mexico hospital staff (N = 2860) [46]. This can possibly be explained by the fact that few studies had used FCV-19S as measurements only focusing on healthcare workers at that time. Overall, the present study found that the low-risk nurses had a higher level of psychological distress, which was different from the results of several previous studies, even during other pandemics [7, 47]. Acceptance of work-related risks was seen among hospital employees and an altruistic acceptance of risk was found to be negatively associated with stress symptoms [48]. These findings suggest that altruistic beliefs may buffer the severity of fear in the high-risk group since most of the nurses were working there voluntarily.

Meaning of life was another focus of study during the life-threatening COVID-19 pandemic. Cox et al. reported that the meaning of life was enhanced, in turn, after being heightened by realizing other benefits (e.g., gratefulness, patience) [26]. The concept of “demoralization” has been mostly applied to patients with major illness, especially advanced cancer patients for decades. Demoralization was defined as anhedonia, an inability to feel pleasure and therefore distinct from depression [49]. Demoralization was considered to be of clinical importance because it was linked to a more precise prediction of suicide risk than depression [49]. Interventions for those affected by demoralization relied on cognitive adjustment rather than sole treatment with anti-depressants [49]. This echoed the two core symptoms in PTSD—negative emotional or cognitive symptoms—newly listed in the Diagnostic and Statistical Manual of Mental Disorders, Fifth edition (DSM-5) [50]. After feeling fearful, individuals underwent a memory process and disrupted cognitive activities before exhibiting PTSD [51]. Many PTSD associated theories indicated that the memory forming pathways in PTSD patients are different from those of unaffected people. For example, Brewin et al. (1996) proposed a dual representation theory, indicating two types of traumatic memory processing [52]: one type was the verbally accessible memory retrieved after a visual stimulus, and the other was the situationally accessible memory under associated situation cues. Working and surviving within the pandemic was like a stressful and life-threatening situation that could facilitate the formation of fear and retrieve traumatic memories among medical staff. The present study population showed that mean scores of the demoralization scale for both the whole group and low-risk group exceeded the cut-off point. Fig 2A shows that demoralization significantly mediated the relationship between fear of COVID-19 and PTSD in the high-risk group. Whereas, in the low-risk group, the mediation effect came from both the fear of COVID-19 and demoralization. Subjective incompetence embedded in cognition was described as the key to demoralization [53]. Although results of the present study may be limited since symptoms of demoralization could overlap with fear, which also caused disability [53]. Li et al. reported that nurses (N = 154) had a higher demoralization level than physicians (N = 417) in a Northern Taiwan medical center [54]. Likewise, nurses working in higher infection risk areas may have more exposure to COVID-19 which required more knowledge and courage to face those challenges. The more confident nurse were about their competencies, the more tasks they could undertake. However, demoralization of the low-risk group could not account for complete mediating effect on the PTSD outcome. Other mediating factors may exist in the low-risk group. All patients admitted to the hospital during the pandemic received COVID-19 tests in advance and patients with positive tests were sent to COVID-specific wards for quarantine and further treatment. However, patients in low-risk areas may still potentially have COVID-19 due to the probabilities of false negatives. Nurses working in low-risk areas were pressured by the uncertain risk of contagion. Contact with these patients when nurses had limited personal protective equipment also influenced fear and uncertainty [8]. Lower preparedness in contrast with high-risk group nurses was another factor. Being relatively neglected compared to receiving greater attention in the high-risk group may also be disturbing [8].

However, despite individual experiences of fear, keeping quality of life stable under challenging circumstances was an essential issue, especially in healthcare workers during the pandemic from an administrative perspective. In the present study, DASS, depression, anxiety and stress showed no between-group differences. These results were consistent with those of a study in Belgium displaying that depression, anxiety, and stress levels did not vary between healthcare working positions [8]. Regarding higher levels of burnout in the low-risk group, nurses may have more chances of directly facing doubts from patients or family, whereas patients in high-risk areas were often separated from others or not that energetic, possibly leading to a higher level of burnout, particularly personal burnout and client-related burnout. Additionally, the present study noted poorer teamwork, subscales of team structure, leadership, and situation monitoring in the low-risk group. In comparison with the pandemic in other countries, the COVID-19 outbreak in Taiwan occurred one year later that the declaration of pandemic. This may suggest that nurses in the high-risk group were well-equipped and had more COVID-19 patient care experience throughout the previous one year. Since the pandemic seemed uncertain, the speed to respond would be a key [55]. Leaders with the ability to anticipate future trends, a stable team structure and sufficient patient care skills may be more indispensable early in the crisis than communications and mutual support from colleagues. Concerning the quality of life, physical, psychological and environmental domains were notably poorer in the low-risk group, whereas social relationships did not differ between the groups. Worries about contagion, subsequent treatment and outcomes, and lack of direct COVID-19 patient care experience may help to explain impaired quality of life among nurses. As for social relationships, since electronic devices have been widely used in the digital era, it was possible for the working population to keep in touch with the network through video, which reduced potential differences between groups. Burnout during the pandemic was derived from intense workload or demands, emotional exhaustion or inadequate resources [8]. In addition to burnout, teamwork was a factor associated with healthcare workers’ job satisfaction and quality of life [56, 57]. Administrators could deal with burnout and teamwork to enhance nurses’ well-being. Therefore, this study explored factors associated with nurse’s quality of life. As shown in Fig 3, both the burnout level and teamwork performance mediated the relationship. The mediation model emphasized the importance of modulating nurses’ workload, working environment, and taking measures to promote team effectiveness, which was intended to help improve nurses’ well-being. It is essential for hospital administrators to notice nurses’ depression, anxiety or stress levels as early as possible and take measures in advance such as adjusting their work content or working environment since their quality of life may, in turn, affect the quality of patient care [58].

### Limitation

There were some limitations in this study. First, convenience sampling in a single medical center was used, which lacks generalizability to nurses across Taiwan or even the world. Second, larger sample size should be considered regarding to total population of nurses in the hospital. Third, we did not examine the baseline pre-pandemic scores and were therefore short of comparable data due to a cross-section study design. The causal relationship between COVID-19 outbreak and mental outcome thus could not be assumed. Also, the relationships between variables were not examined separately in the low- or high-risk group since the sample size was not large. However, these relationships may vary by different levels of risk of infection. The parallel mediation effect should be examined in a larger sample and with longitudinal design since a significant correlation was found even though beta was not high between the two mediators. Lastly, non-response bias commonly seen in online surveys were noticed [59], including that answers to survey questions needed to be typed instead of just clicking on buttons.

## Conclusion

This study indicated nurses working in hospital are at risk of developing PTSD symptoms during a pandemic such as the COVID-19 pandemic. Identification of predictors such as fear symptoms and demoralization may be helpful for designing specific intervention programs for them to assist nurses in recovery. Adequate training and preparedness may regulate the fear and subsequent memory formation before PTSD develops. In addition, introducing administrative measures such as reducing burnout level and enhancing teamwork effectiveness may help to restore quality of life among nurses during a crisis situation such as COVID-19. Future longitudinal follow-up studies about their mental outcomes and resilience are warranted.

## Supporting information

S1 File (PDF)
