# Supplementary material for: Factors associated with PTSD symptoms and quality of life among nurses during the COVID-19 pandemic: A cross-sectional study
Source: PLoS One. 2023 Mar 24;18(3):e0283500. doi: 10.1371/journal.pone.0283500 (PMC10038253; doi:10.1371/journal.pone.0283500)
Supplement: S1 File — (PDF) [file pone.0283500.s001.pdf]

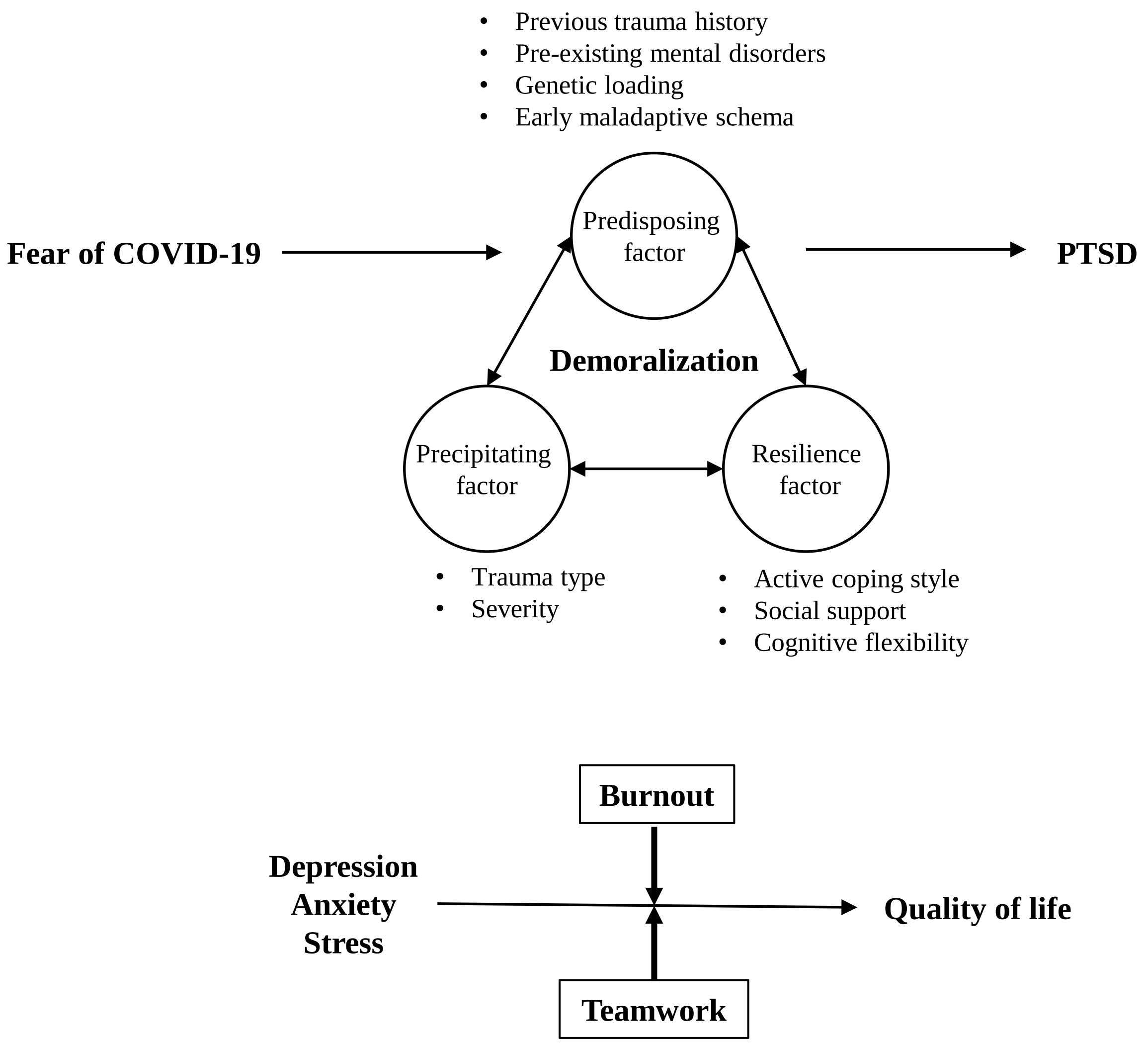

S1 Appendix 1. Framework of the study

S2 Appendix 2. Pearson’s correlations before parallel mediation

	**CBI total score**	**TPOT total score**	**WHOQOL-BREF total score**
**DASS**	β=0.633***	β=-0.209***	β=-0.531***
**CBI total score**		β=-0.272***	β=-0.543***
**TPOT total score**			β=0.443***

DASS = Depression, Anxiety and Stress Scale; CBI = Copenhagen Burnout Inventory; TPOT = Team Performance Observation Tool

$p < 0.05$*, $p < 0.01$**, $p < 0.001$***
